# Novel 1,2-Bismethacrylate-3-Eugenyl Propane for Resin Composites: Synthesis, Characterization, Rheological, and Degree of Conversion

**DOI:** 10.3390/polym15061481

**Published:** 2023-03-16

**Authors:** Haifa Masfeer Al-Kahtani, Abdel-Basit Al-Odayni, Waseem Sharaf Saeed, Ali Robaian, Abdullah Al-Kahtani, Taieb Aouak, Ali Alrahlah

**Affiliations:** 1 Chemistry Department, College of Science, King Saud University, P.O. Box 2455, Riyadh 11451, Saudi Arabia; 2 Engineer Abdullah Bugshan Research Chair for Dental and Oral Rehabilitation, College of Dentistry, King Saud University, Riyadh 11545, Saudi Arabiaaalrahlah@ksu.edu.sa (A.A.); 3 Department of Chemistry, Faculty of Education, Thamar University, Dhamar 87246, Yemen; 4 Conservative Dental Sciences Department, College of Dentistry, Prince Sattam Bin Abdulaziz University, Alkharj 11942, Saudi Arabia; 5 Restorative Dental Sciences Department, College of Dentistry, King Saud University, Riyadh 11545, Saudi Arabia

**Keywords:** eugenol, eugenol derivative, dimethacrylated derivative, resin, dental composite, rheology, degree of conversion

## Abstract

This work aimed to synthesize a novel dimethacrylated-derivative of eugenol (Eg) (termed EgGAA) as potential biomaterial for certain applications such as dental fillings and adhesives. EgGAA was synthesized through a two-step reaction: (i) a mono methacrylated-eugenol (EgGMA) was produced via a ring-opening etherification of glycidyl methacrylate (GMA) with Eg; (ii) EgGMA was condensed with methacryloyl chloride into EgGAA. EgGAA was further incorporated in matrices containing BisGMA and TEGDMA (50:50 wt%) (TBEa), in which EgGAA replaced BisGMA as 0–100 wt% to get a series of unfilled resin composites (TBEa0–TBEa100), and by addition of reinforcing silica (66 wt%), a series of filled resins were also obtained (F-TBEa0–F-TBEa100). Synthesized monomers were analyzed for their structural, spectral, and thermal properties using FTIR, ^1^H- and ^13^C-NMR, mass spectrometry, TGA, and DSC. Composites rheological and DC were analyzed. The viscosity (*η*, Pa·s) of EgGAA (0.379) was 1533 times lower than BisGMA (581.0) and 125 times higher than TEGDMA (0.003). Rheology of unfilled resins (TBEa) indicated Newtonian fluids, with viscosity decreased from 0.164 Pa·s (TBEa0) to 0.010 Pa·s (TBEa100) when EgGAA totally replaced BisGMA. However, composites showed non-Newtonian and shear-thinning behavior, with complex viscosity (η*) being shear-independent at high angular frequencies (10–100 rad/s). The loss factor crossover points were at 45.6, 20.3, 20.4, and 25.6 rad/s, indicating a higher elastic portion for EgGAA-free composite. The DC was insignificantly decreased from 61.22% for the control to 59.85% and 59.50% for F-TBEa25 and F-TBEa50, respectively, while the difference became significant when EgGAA totally replaced BisGMA (F-TBEa100, DC = 52.54%). Accordingly, these properties could encourage further investigation of Eg-containing resin-based composite as filling materials in terms of their physicochemical, mechanical, and biological potentiality as dental material.

## 1. Introduction

Recently, (meth)acrylated-eugenol derivatives have drawn considerable interest, particularly in fields requiring immobilized eugenol moiety for long-term functioning [1,2]. Eugenol (4-allyl-2-methoxyphenol) is a versatile, naturally occurring bioactive compound, abundantly available either naturally or synthetically. It is also an attractive aromatic building block for novel bio–based monomers [1]. Despite the presence of a vinyl group in its chemical structure, it is of allyl type and, thus, electron resonance (charge or radical) stabilization restricts polymerization advancement under conventional conditions. Nevertheless, it has a long history of beneficial use in medicine and food and functions as a gentle local anesthetic, antiseptic, and antimicrobial agent at controlled dosages [3]. Due to such beneficial features, it is widely engaged in many industrials, such as cosmetics, pharmaceuticals, and dentistry [4], since it is an important precursor for a number of transformations including adhesives, polymerizable, and non-polymerizable derivatives.

To modify eugenol, a number of reaction sites could be targeted, of which allylic and phenolic groups are common. Phenolic hydroxyl, in particular, is a target reaction center for synthesis of a number of esters- and ether-derivatives of eugenol. In this course, polymerizable derivatives, predominantly of (meth)acrylate type, have received a special interest over others, due to the demand of an immobilizable, moldable, bioactive substance for sit-setting fabrication with durability and long-life functioning [5,6]. The reported (meth)acrylated-eugenol monomers were either mono-, di-, or multifunctional ones, being investigated for their potential application as adhesives, dental filling, and orthopedic cements [6,7,8,9]. They constitute an important class of eugenol (Eg) derivatives due to their potential benefits for orthodontic and dental applications and as prospective adhesives.

Eugenol-glycidyl methacrylate (EgGMA)—3-eugenyl-2-hydroxypropyl methacrylate—is a mono-functional monomer which, upon polymerization, the Eg-moiety pendant group may affect polymerization progression and further, driven certain properties of the end-product. Its application as a resin additive [8,9] has been reported, and the primary evaluation indicated biocompatibility; however, its bioactivity feature is still an open subject. The structure properties for Eg may be retained, as hinted by various researchers [2,10]. The advantage of multi-(meth)acrylated monomers over mono-monomers is that, after polymerization, the product from the former typically has a stronger three-dimensional network structure as a result of crosslinking reactions. Therefore, multi-functional monomers may be more useful for certain application such as adhesion and dental fillings.

Dental resin composites suffer from polymerization shrinkage, resulting in bacterial microleakage and demineralization at tooth-composite margins, leading to secondary caries [11,12] that affect the longevity of composite restorations. A remedy to this problem would be advanced through remineralizing the hard tissues near the restoration or developing antibacterial composites [11]. However, incorporating ion-releasing fillers such as hydroxyapatite, which supports preventing secondary caries by remineralization, may reduce the composite strength. Furthermore, adding releasable antibacterial agents such as silver- or zinc-containing particles, antibiotics, and chlorohexidine may solve the problem; however, the method suffers from the rapid initial release of the agents, thus resulting in short-term effectiveness. Hence, the emerging idea was to apply immobilized active agents within the restorative composite [13]. It is reported that the mechanical performance is reduced accordingly; therefore, the amounts of immobilizable agents (i.e., active monomers) should be controlled well. Therefore, before proceeding further, a preliminary assessment of their physical, chemical, rheological, and reactivity properties may be necessary.

In this work, 3-(4-allyl-2-methoxyphenoxy) propane-1,2-diyl bis(2-methylacrylate) (termed EgGAA) was synthesized and fully characterized for its structure. Some of its physicochemical and thermal properties were also investigated. Moreover, a series of resin mixtures containing bisphenol A-glycidyl methacrylate (BisGMA) and triethylene glycol dimethacrylate (TEGDMA) as conventional matrices in resin-based dental composites were prepared. The effect of BisGMA replacement by EgGAA in the of range 0–100 wt% was assessed in terms of the degree of conversion and rheological properties. It is hypothesized that (1) the newly synthesized EgGAA monomer has a lower viscosity than the conventional BisGMA monomer, (2) the replacement of BisGMA by EgGAA in the matrix would not affect the viscosity of the composite, and (3) such replacement would not significantly impact the DC of the composite.

## 2. Materials and Methods

### 2.1. Materials

Eugenol (Eg, 98.5%), glycidyl methacrylate (GMA, 98%), methacryloyl chloride (MAC, 97%), camphorquinone (CQ, 97%), BisGMA (>98%), TEGDMA (>95%), tetraethyl orthosilicate (TEOS, 99%), 2-(dimethylamino)ethyl methacrylate (DMAEMA; 98%), (3-(trimethoxysilyl)propyl methacrylate (γ-MPS, 98%), hydroquinone (HQ, >99%), triethylamine (Et_3_N, >99%), and ethanol absolute (EtOH, ≥99.8%), were obtained from Sigma-Aldrich (Taufkirchen, Germany). Triphenylphosphine (Ph_3_P, 99%) was purchased from Cica-reagent (Kanto Chemical, Tokyo, Japan). Hexane (Hx, 95%) was purchased from Avonchem (Macclesfield, UK). Ethyl acetate (EA, ≥99.5%), ammonium hydroxide (NH_4_OH, 35%), sodium sulfate anhydrous (Na_2_SO_4_, >99%), and dichloromethane (DCM, 99%) were purchased from Fisher Scientific (Loughborough, England, UK). Sodium hydroxide pellets NaOH (98%) was obtained from Alfa Aesar (Karlsruhe, Germany). All materials were used as received.

### 2.2. Synthesis of EgGAA

**Figure 1:**
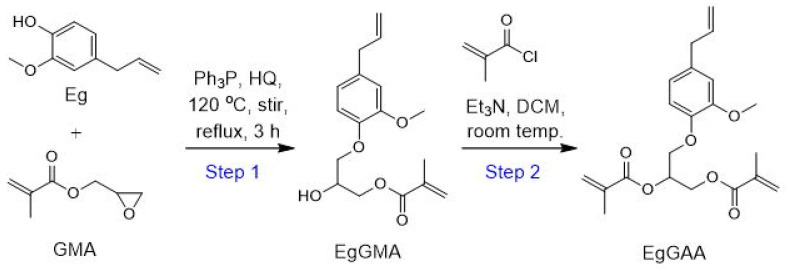
Synthesis of EgGAA monomer.

EgGAA was synthesized through a two-step protocol, as shown in Figure 1.

Step (I). Synthesis of EgGMA. The mono-methacrylated eugenol-derivative (EgGMA) was synthesized following a previously reported method [14]. Briefly, in a three-necked round bottom flask, equimolar amounts of the starting materials, Eg and GMA, were mixed by stirring. The reaction setup was built with a refluxing system at 120 °C, N_2_ gas streaming as an inert environment, and magnetic stirring for ensuring homogeneity and an even temperature distribution. To this mixture, 0.5 and 1.0 wt%, with respect to the total monomers Eg and GMA, of HQ and Ph_3_P, respectively, were added. The mixture was stirred at room temperature until complete dissolution of the components (about 10 min). After that, the reaction mixture was heated at 120 °C for 3 hrs, brought to room temperature, and left to stir overnight. The mixture was filtered to remove the solid Ph_3_P oxide by-product and adequately washed with EA/Hx (3:7 *v*/*v*). The product was concentrated using rotary evaporator at 40 °C, then subjected to liquid column chromatography (LCC) purification as described below.

Step (II). Synthesis of EgGAA. The dimethacrylated eugenol (EgGAA) was synthesized via a condensation reaction between EgGMA (from step I) and methacryloyl chloride (MAC) in the presence of triethylamine (Et_3_N) as a catalyst base: this method was adapted from the literature [7,15] with slight modification. Hence, EgGMA and Et_3_N (1:1.2 molar ratio) were dissolved in a dry dichloromethane (DCM) and then introduced into a three-necked, round-bottomed flask. The reaction environment was filled with N_2_ inert gas. Then, an excess (equal to Et_3_N) of MAC previously diluted in DCM (50 *v*/*v*%) was added dropwise at a slow rate with constant stirring at room temperature. The reaction mixture was kept stirred at room temperature for 24 h under a N_2_ atmosphere. After that, the reaction mixture was filtered to remove Et_3_N–hydrochloride and washed several times with NaOH (5%) and water to remove unreacted reagents. The eluted liquid phase was dried using anhydrous Na_2_SO_4_ and the solvent was removed under reduced pressure using a rotary evaporator apparatus at 40 °C. The obtained oily liquid was further purified using LCC.

### 2.3. Purification of Synthesized Monomers

The synthesized monomers, EgGMA and EgGAA, were purified using the typical chromatography process. Hence, a thin-layer chromatography (TLC; an aluminum silica gel plate of 0.1 mm thickness) was used for preliminary screening of the mixture, purity verification, and for mobile phase investigation. The appropriate mobile phase for TLC, which applied for LCC afterwards, was determined by using various ratios of EA (polar) and Hx (nonpolar) solvent mixtures, from 10 to 90 vol%. The appropriate separation with adequate retention factor was selected for further LCC. Thus, the EA/Hx solvent ratios of 3:7 and 1:9 were found to be optimal for EgGMA and EgGAA purification, respectively. The isolated pure monomers were stored and cooled in a refrigerator (~8 °C) until use.

### 2.4. Synthesize of Silanized Silica

The target silanized nanosilica (S-SiO_2_) was synthesized following the well-known Stöber method [16]. Therefore, a solution of 40 mL water, 250 mL absolute ethanol, and 25 mL ammonium hydroxide was prepared, cooled in an ice bath. Then, 45 mL of TEOS was added dropwise for 5 min. The reaction mixture was brought to room temperature and a second portion of 33 mL TEOS in 250 mL EtOH was added and stirred for 8 hrs. Subsequently, the organosilane (γ-MPS; 10 vol% with respect to TEOS) was added, and the mixture was left to stir overnight. The obtained S-SiO_2_ was separated with centrifugation. It was then washed three times with EtOH using a repeated suspension-centrifugation protocol followed by solvent removal using a rotary evaporator. Finally, the obtained S-SiO_2_ were dried under vacuum overnight.

### 2.5. Preparation of Unfilled and Filled Resins

For rheological property analysis, the viscosity of the synthesized monomer (EgGAA) was measured and compared to that of the conventional monomers commonly used in resin-based dental composites including BisGMA and TEGDMA. The matrices to be analyzed were formulated as given in Table 1, without initiation system, in which BisGMA was replaced by EgGAA (as 0, 25, 50 and 100 wt%). TEGDMA was used as a diluent (50 wt% of each matrix and kept constant). Typically, in four dark vials, the target series of resins (TBEa0–TBEa100) were manually mixed until clear homogeneous mixtures were obtained. Then, composites were prepared by mixing the above resins with synthesized nano-silica as 34:66 wt%, respectively. For DC, an initiator system of 1 wt% per matrix (0.2 wt% CQ and 0.8 wt% DMAEMA) was initially dissolved in the monomeric portion before adding of silica fillers; then, it was treated as above. Such sensitive materials (monomers, resins, and composites) were kept in dark containers at ~8 °C until used.

### 2.6. Monomer Characterization

FTIR spectra were obtained using a Nicolet iS10 spectrophotometer from Thermo-Scientific (Madison, WI, USA), equipped with an attenuated total reflection (ATR) accessory on the range of 650–4000 cm^−1^, averaged from 32 readings with 4 cm^−1^ resolution. The ^1^H- and ^13^C-NMR profiles were recorded on a JEOL Delta-NMR spectrometer (JOEL Resonance, Tokyo, Japan) at 400 MHz, using deuterated chloroform (CDCl_3_) as the solvent and tetramethylsilane as the internal standard. Delta v5.0.4 software (JEOL, Tokyo, Japan) was employed for data visualization. Mass fragmentation patterns were acquired using a JMS-T100 LP ToF-MS spectrometer (JEOL, Tokyo, Japan) equipped with a DART ion source (IonSense, Saugus, MA, USA) and operated in the positive-ion mode. Fragments were obtained at 300 °C, and selected peaks were designated using the MassCentre software (v.1.3.m, JEOL, Tokyo, Japan) against PEG200 for 1.55–2.00 min. Thermogravimetric (TGA) measurement of EgGAA was carried out on a Mettler-Toledo TGA/DSC 1 Star system (Columbus, OH, USA). Material of about 12 mg was applied into the cell and heated from 25 to 800 °C at a heating speed of 10 °C/min under a nitrogen gas flow of 20 mL/min. Differential scanning calorimetry (DSC) curves were acquired on a Shimadzu DSC 60A (Kyoto, Japan) for 10–12 mg samples and heated from −120 to 50 °C at a heating rate of 10 °C/min under a nitrogen atmosphere controlled at 50 mL/min.

### 2.7. Rheology Test

An MCR-72 Anton Paar rheometer (Anton Paar, Graz, Austria) was used for viscosity and flow behavior assessment of the monomers (BisGMA, EgGAA, TEGDMA, and UDMA) and their unfilled and filled resins. Viscosity of resins (monomers and their mixtures) was analyzed using a rotational mode over a steady shear sweep of 0.01–1000 s^−1^, employing 25-mm parallel plates and a 0.25-mm measuring gap. Due to machine limitation and a huge viscosity range variation between investigated monomers, recording was limited to the linear region. The rheological properties of the filled resins (experimental composites) were determined using the oscillatory mode over a frequency sweep of 0.1–100 (ω, rad/s). The machine Peltier plate-temperature controlling system was set at 25 ± 0.1 °C during all measurements. All materials were scanned at least 3 times (*n* = 3) and values were averaged and reported.

### 2.8. Degree of Conversion Test

The degree of conversion (DC) of the prepared composites F-TBEa was determined using the ATR-FTIR technique (FTIR, Nicolet iS10) [17]. For this, the specimen (*n* = 5 replicates) to be measured was pressed in a fabricated stainless-steel disk-shaped mold (5 × 1 mm), which was previously mounted on the ATR crystal stage. The side to be cured (the top side) was covered with a plastic strip and a glass slide to prevent the oxygen species inhibitory effect on polymerization and to flatten and avoid air trapping. The spectrum for the uncured sample was recorded; then, it was irradiated for 60 s from the top side using a LED light curing unit (Bluephase, Ivoclar Vivadent, Schaan, Liechtenstein) with 650 mW/cm^2^, as verified by an Optilux Radiometer (Kerr Corp., Danbury, CT, USA), with a wavelength range of 385 to 515 nm and a 10-mm rotatable tip. The tip was set in direct contact with the specimen surface. After 10 min of aging, the spectrum was recorded (cured sample).

The DC was calculated based on the difference between the mole ratio of C=C stretching peaks of vinylic at 1638 cm^−1^ and bending vibration of C-H at 1451 cm^−1^, before and after curing [18]. Thus, the areas of the target peaks were procured from the corresponding spectra and utilized for DC calculation, according to Equation (1).

(1) $$DC(\%)=\left[1-\frac{{\left(\frac{A_{1638}}{A_{1451}}\right)}_{cured}}{{\left(\frac{A_{1638}}{A_{1451}}\right)}_{uncured}}\right]\times 100$$

where A_1638_ and A_1451_ were the areas under the peaks at 1638 and 1451 cm^−1^ for C=C stretching and C-H bending, respectively.

### 2.9. Statistical Analysis

Data from rheology and DC experiments were statistically analyzed using one-way analysis of variance (ANOVA) and Tukey post-hoc tests (SPSS 21 (IBM Corp., Armonk, NY, USA). A *p*-value < 0.05 was considered significant.

## 3. Results and Discussion

### 3.1. Synthesis of Monomers

The target EgGAA monomer was synthesized through a two-step protocol, as shown in Figure 1. In the first step, eugenol-derived mono-methacrylate EgGMA was synthesized from its precursors, Eg and GMA, resulting in an oily light-yellow product (isolated yield, ~56%). In the second step, EgGAA was synthesized via a catalyzed condensation reaction between EgGMA and MAC. The obtained EgGAA was lighter in its yellowish color than EgGMA, and the yield was about 39%. The reaction mechanisms were illustrated with details in the supplementary information (Section S1 and Figure S1).

### 3.2. Spectral Analysis

#### 3.2.1. FTIR

**Figure 2:**
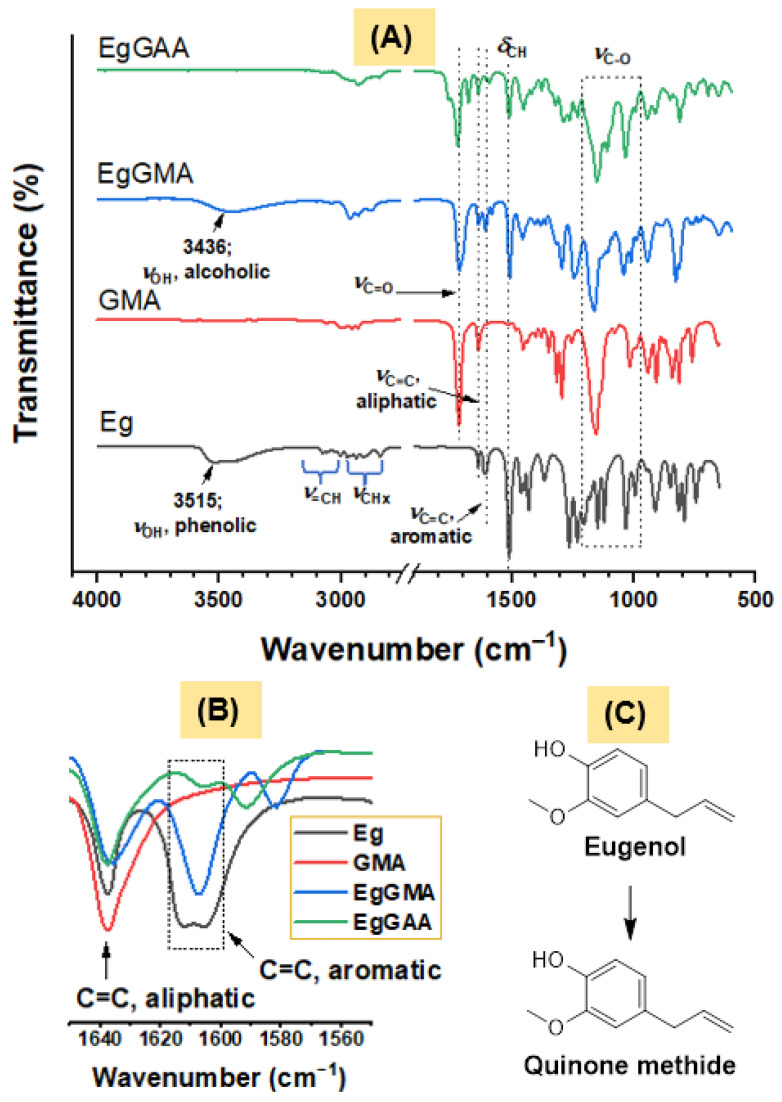
(**A**) FTIR spectra of Eg, GMA, EgGMA and EgGAA. (**B**) Spectral range of C=C bonds. (**C**) Eugenol and quinone methide structures.

The chemical structures of EgGMA and EgGAA were confirmed by FTIR spectroscopy. The spectra of Eg, GMA, EgGMA, and EgGAA are gathered in Figure 2A, and the corresponding characteristic bands are given in Table S1. The spectra of Eg, GMA, and EgGMA are in good agreement with the literature [14]. Hence, EgGMA was synthesized from its commercially available products, Eg and GMA. The successful production of EgGMA was confirmed via the disappearance of Eg phenolic–OH peak at 3515 cm^−1^ and the emergence of secondary alcohol hydroxyl (EgGMA–OH) at 3436 cm^−1^. Furthermore, the peak characterizing the epoxy ring in GMA at 907 cm^−1^ was moved to 914 cm^−1^ in EgGMA, indicating ring opening [19,20,21]. By monitoring peaks of aliphatic and aromatic C=C bonds at 1638 and 1606 cm^−1^ [22], the structural integrity of EgGMA can be confirmed. The lonely peak seen in Eg spectra at 1612 cm^−1^ is attributed to the quinomethide structure of Eg that is difficult to be generated in EgGMA (or EgGAA) (Figure 2B,C).

The successful synthesis of EgGAA can be affirmed by the disappearance of the alcohol-OH peak at 3436 cm^−1^. Structurally, EgGAA holds the same chemical bonds as EgGMA and, thus, similar spectra are expected. However, the former has two methacrylate moieties; therefore, its molecular weight (374.43 g/mol) is higher than EgGMA (306.36 g/mol). The newly observed peaks in the spectrum of EgGAA at 1759 (shoulder) and 1677 cm^−1^ could be assigned to C=O located at different geometries and caused by its high molecular weight and ester groups; the C=O main peak was observed as a strong band at 1721 cm^−1^. This possibly different structural configuration results from the isoeugenol structure and primary/secondary alcoholic EgGMA–OH; it is worth noting that these peaks were not diminished after polymerization of the monomer (results not shown). Other bond vibrations are given in Table S1.

#### 3.2.2. NMR Analysis

**Figure 3:**
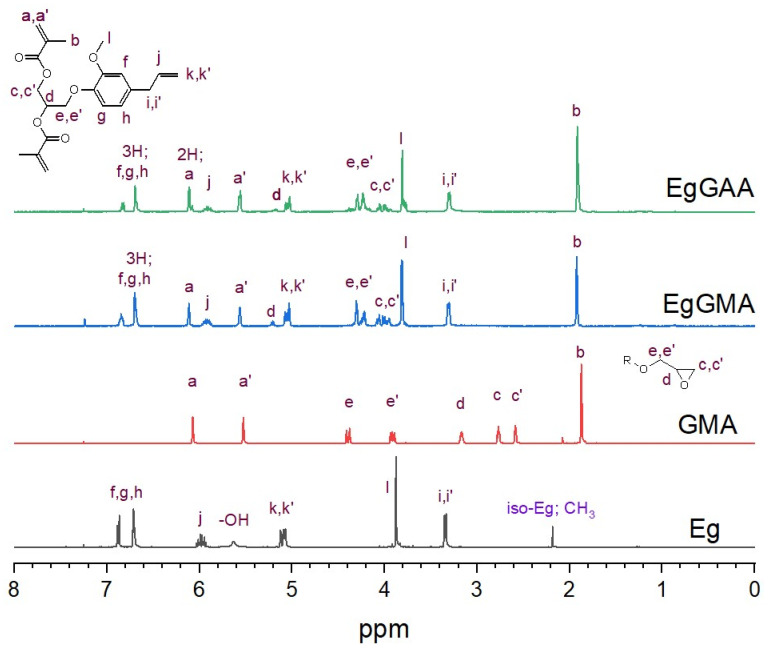
^1^H NMR spectra of Eg, GMA, EgGMA, and EgGAA. Peaks were marked by letters as defined in the included structure.

Figure 3 shows the ^1^H NMR spectra for materials under investigation, i.e., the precursors (Eg, GMA, and EgGMA) and the target product (EgGAA). As seen in this figure and Table S2, all protons in the molecules were identifiable. By correlation between peak integration and the number of protons, the structural integrity of the products was confirmed. Bands corresponding to Eg, GMA, and EgGMA were compared with the literature [7,14,23,24]. The spectral pattern of EgGAA is close to that of EgGMA, due to their similarity. Nevertheless, the characteristic peaks, specifically the ones close to the reaction center (c, d, and e protons), were slightly downfield shifted due to the deshielding difference of alcohol– and ester–oxygen atoms. For example, peaks of protons around the condensation center (c, c′, d, e and e′) in EgGMA monomer observed at 4.12–3.91 (c and c′), 5.21 (d), and 4.37–4.15 (e and e′) were shifted to 4.31–3.93, 5.71, and 4.31–4.20 ppm, respectively, while shifts on remote protons were smaller (Table S2).

**Figure 4:**
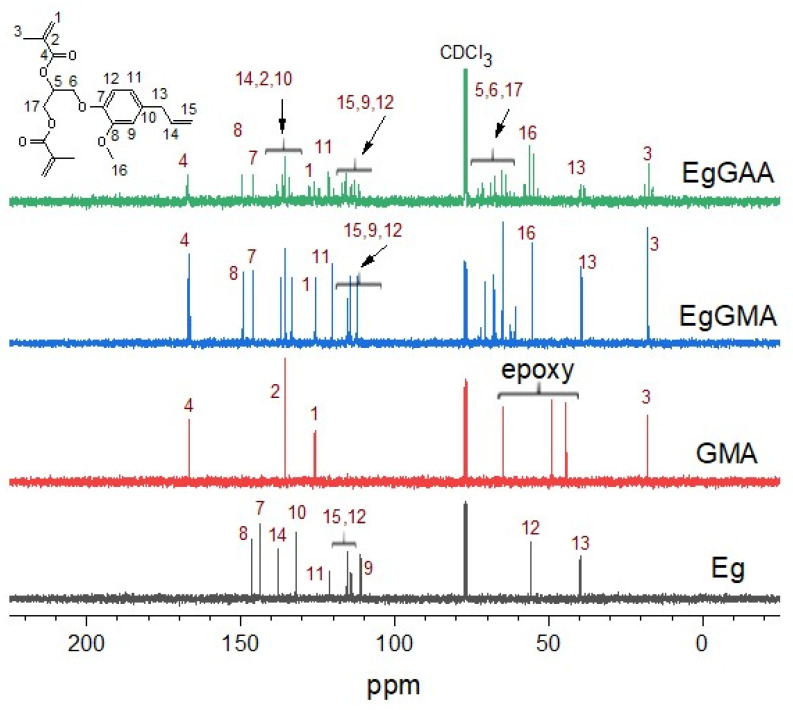
^13^C NMR spectra of Eg, GMA, EgGMA, and EgGAA. Peaks were numerically marked as in the included structure.

The ^13^C NMR spectra are presented in Figure 4, and peaks are assigned in Table S2. The spectral profiles of Eg, GMA, and EgGMA were in accordance with the literature [7,14,23,24]. By inspection of the EgGAA spectrum, all carbons can be identified, with insignificant chemical shift differences compared to EgGMA. It could also be seen that the shift in C-5 (reaction center) peak, from 67.68 (EgGMA) to 68.96 (EgGAA) ppm, is detectable. On the other hand, traces of low intensity peaks were observed, which possibly originated from iso-structures of methacrylated Eg-derivatives (EgGMA and EgGAA).

#### 3.2.3. Mass Analysis

**Figure 5:**
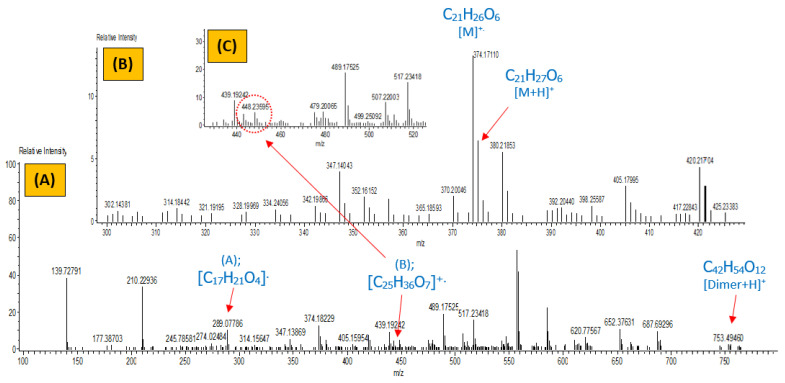
Mass spectra of EgGAA monomer: (**A**) full fragmentation pattern, (**B**) molecular ion region, and (**C**) peak-B region.

To further confirm the chemical structure of the synthesized EgGAA, the mass spectrum was also recorded and the corresponding possible fragmentation route was predicted, as shown in Figure 5 and Figure S2. The observed and calculated *m*/*z* of the EgGAA molecular ion are compared in Table S3, along with mass differences and unsaturation degree. The peak of the parent ion ([M]^+^; C_21_H_26_O_6_) and the protonated one ([M+H]^+^; C_21_H_27_O_6_) were clear, at *m*/*z* values of 374.17 and 375.18, respectively. Furthermore, the fragmentation pattern, obtained under mass analysis conditions, suggests oligomerization with a dimer ([Dimer+H]^+^; C_42_H_54_O_12_) fragment being the more visible one at *m*/*z* of 753.49. According to Figure S2, the dimer may also decompose into peak-B ([C_25_H_36_O_7_]^+^, *m*/*z* = 448.24). Moreover, a fragment of methacrylate-truncated EgGAA was also seen at *m*/*z* of 289.08 (peak-A; ([C_17_H_21_O_4_]^+^)). These results confirm the success of EgGAA synthesis, supporting its molecular formula and possible structure mass fragmentation.

### 3.3. Thermal Analysis

#### 3.3.1. TGA Analysis

**Figure 6:**
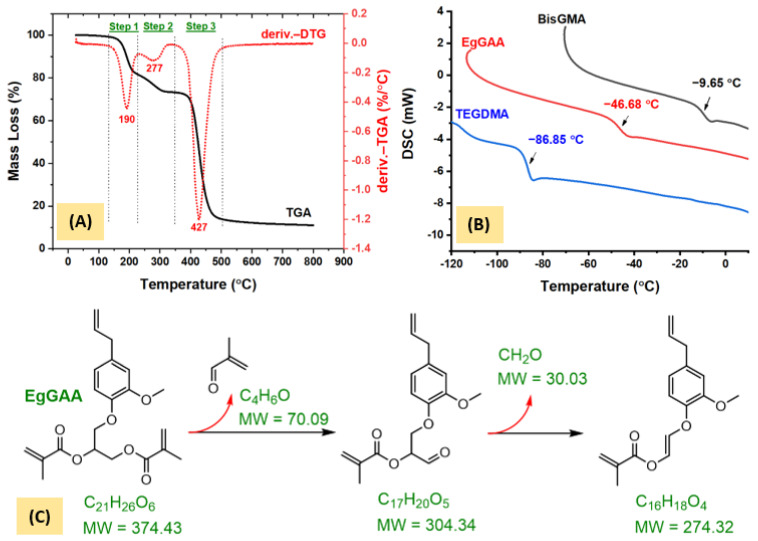
TGA/DTG (**A**) and DSC (**B**) curves and predicted thermal mass loss (**C**) of EgGAA monomer decomposition.

The TGA thermograms and the predicted decomposition steps of EgGAA are given in Figure 6A,C and Table S4. The curve shows a stepwise decomposition, distinctly consisting of three steps observed at DTG of 190, 277, and 427 °C. The steps are assigned to breaking down of one methyl methacrylate moiety—initiated by removal of C_4_H_6_O segment (Step 1)—followed by CH_2_O (Step 2), with experimental mass losses of 18 and 9 wt% (total = 27 wt%) as compared to predicted values of 18.7 and 8.1 wt% (total = 26.8 wt%), respectively. These observations are typical for a number of dimethacrylated monomers, as reported by Bannach et al. [25]. The authors concluded that the main volatiles are methacrylic acid, 2-hydeoxymethylmethacrylate, formic acid, or their mixtures. In the current study, methyl methacrylate was first detected below 300 °C, which is in close agreement with reference [25]. The last step (Step 3) is the evaporation of the major part of the molecule with a mass loss of about 60 wt%, leaving a residual mass of 11 wt% consisting of three atoms of carbons at 800 °C. This result may indicate early thermal decomposition of EgGAA under the applied condition of an inert environment of nitrogen gas, heating speed of 10 °C/min, and heating range of 25–800 °C.

#### 3.3.2. DSC Analysis

The DSC curve of EgGAA was compared to that of BisGMA and TEGDMA in Figure 6B. The glass transition temperature (*T*_g_) was reported as the midpoint of the sigmoidal change in the heat capacity [26], Table S5. The *T*_g_ value of EgGAA was found at −46.7 °C, less than that of BisGMA at −9.7 °C, but higher than that of TEGDMA at −86.85 °C; the *T*_g_ values of BisGMA and TEGDMA were reported at about −7 and −83.9 °C [25,27], respectively. The *T*_g_ value depends on the mobility of molecules, which is associated with their chemical structure and type of molecular interactions such as London dispersion forces, hydrogen bonding, and dipolar interaction [28]. In such a context, BisGMA has a strong intermolecular hydrogen bonding—a feature that is not present in EgGAA—and thus, displayed higher *T*_g_.

### 3.4. Rheology Analysis

**Figure 7:**
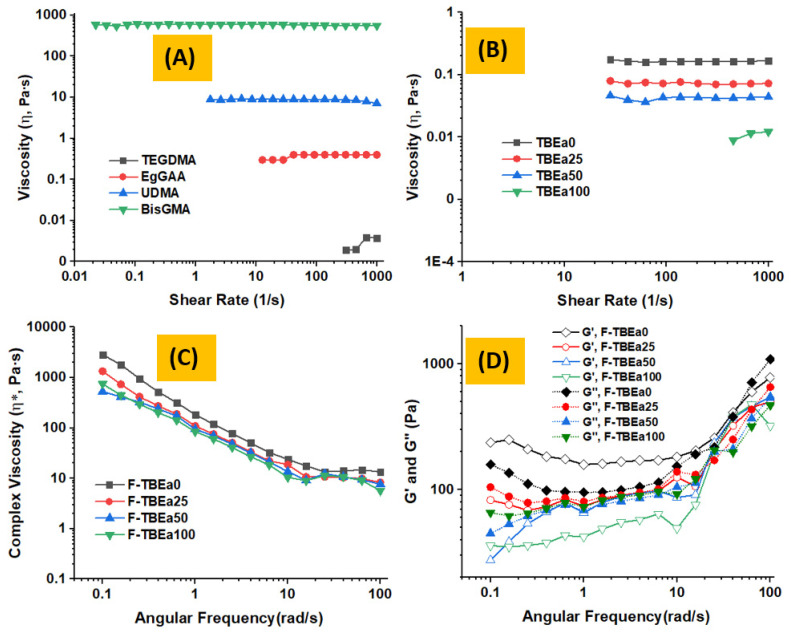
(**A**) Rheometrically analyzed: (**A**) viscosity of monomers (TEGDMA, EgGAA, UDMA, and BisGMA); (**B**) viscosity of resin matrices (TBEa(0–100)), (**C**) complex viscosity of prepared resin composites (F-TBEa(0–100)), (**D**) storage (G′) and loss (G″) moduli of F-TBEa(0–100) composites.

Viscosity is a measure of fluid resistance to flow and is governed by the strength of intermolecular forces as a consequence of the component’s molecular structures, sizes, and shapes. There are two kinds of viscosity: dynamic and kinematic; they are interchangeable when density is known. Dynamic viscosity measures the relationship between shear stress and shear rate, while kinematic viscosity is density-dependent expressed as the ratio of the dynamic viscosity to the density of a fluid. As can be seen in Figure 7A and Table 2, the dynamic viscosity (*η*, Pa·s) of the synthesized EgGAA was 0.379, which is significantly lower than that of BisGMA (581.0) and UDMA (9.106), but higher than that of TEGDMA (0.003); therefore, the first hypothesis was accepted. Compared to other monomers, BisGMA and UDMA have, respectively, two hydroxyl and two amine groups that are able to form strong hydrogen bonds and kinetically cause a reduced degree of molecular freedom. In contrast, TEGDMA and EgGAA contain no OH or NH groups, and therefore, hydrogen bonds cannot be formed. Hydrogen bonds can engage in intramolecular and intermolecular interactions which, in turn, affect monomer configuration and mobility on the molecular level, resulting in increased viscosity. In the comonomer mixtures (TBEa resins), hydrogen bonding could improve compatibility between mixed monomers [29]. The viscosity of the evaluated monomers and their mixtures was measured under similar experimental conditions and, therefore, the viscosity differences can only be attributed to their chemical properties. Besides hydrogen bonds, BisGMA and UDMA have higher molecular masses (512.60 and 470.56 g/mol) and extended chains than TEGDMA (286.32 g/mol) and EgGAA (374.43 g/mol), which may also contribute to their high viscosities. Despite the fact that both TEGDMA and EgGAA have six-member oxygen atoms (two carbonyls, two esters, and two ethers), the higher molecular weight and the rigidity caused by chain substitution of EgGAA may drive, in part, its higher viscosity compared to the TEGDMA monomer (Figure S3).

After mixing, the resulting resins (TBEa0–TBEa100) (Figure 7B) were visibly one phase, indicating compatible contents. Moreover, the TEGDMA diluent has effectively reduced the viscosity of BisGMA from 581.0 to 0.164 Pa·s, when 1:1 (*wt*/*wt*) dilution was applied. Such a huge effect may explain the role of hydrogen bonding in BisGMA, which was disrupted after TEGDMA addition. To investigate the possibility of replacing the base resin (BisGMA) with the lesser viscosity EgGAA monomer, a series of replacements by the latter was carried out, keeping the TEGDMA ratio constant (50 wt% per resin). Compared to TBEa0 (*η*, 0.164 Pa·s), replacement of 25, 50, and 100 BisGMA (i.e., in TBEa25, TBEa50, and TBEa100) by EgGAA have resulted in viscosities that are 2.3, 3.9, and 15.0 times lower than the BisGMA-unreplaced resin (TBEa0); thus, the rejection of the second hypothesis is supported. Compared to neat TEGDMA, TBEa100 is still 3.7 times higher. Interestingly, despite that, a viscosity reduction of only 3.9× was observed when 50 wt% of BisGMA was exchanged with EgGAA (TBEa50); a drop of about 15× (from 0.164 to 0.010 Pa·s) was recorded when BisGMA was entirely replaced by EgGAA (TBEa100).

The viscoelastic behavior of nano-silica-filled resins (F-TBEa0, F-TBEa25, F-TBEa50, and F-TBEa100) was also rheometrically analyzed. The frequency sweep-dependent complex viscosity (η*) is presented in Figure 7C. In this figure, the rheological results showed non-Newtonian and shear-thinning behavior, with η* generally decreasing as replacement of BisGMA by EgGAA was advanced from 0–100 wt% (F-TBEa0 to F-TBEa100). The drop in η* from 174.553 rad/s (F-TBEa0) to 102.726 rad/s (F-TBEa25) is statistically significant; therefore, the second hypothesis is rejection. However, no significant differences between F-TBEa25 and F-TBEa50 or F-TBEa100 were observed. Furthermore, a plateau at frequencies higher than 10 rad/s was observed almost for all composites, indicating frequency-independent η*.

To assess the contribution of the viscous and elastic portions in the viscoelasticity of the composites, the storage (G′) and loss (G″) moduli were also analyzed over the measured angular frequency of 0.1–100 rad/s and depicted in Figure 7D. The profiles of G″ and G′ indicate that the matrix incorporating BisGMA (i.e., F-TBEa0) has the highest values of G″ and G′. The values gradually decreased as the proportion of EgGAA increased. However, the difference between G″ values (and G′ as well) of binary matrix-containing composites (F-TBE0 and F-TBEa100) was high while being noticeably low between the ternary matrix-containing F-TBEa25 and F-TBEa50. For example, the value of G′ at the frequency of 1 rad/s (a demonstrative point) of F-TBEa0, F-TBEa25, F-TBEa50, and F-TBEa100 were 158.3, 74.2, 65.3, and 42.1 Pa, respectively; the corresponding loss factors (tan *δ*) were 0.60, 1.08, 1.05, and 1.74, respectively. The crossover points at which G″ and G′ have equal values were observed at 45.6, 20.3, 20.4, and 25.6 (rad/s) with tan δ of 457, 151, 152, and 208, respectively. It is noted that the difference between G″ and G′ was small for F-TBEa25 and F-TBEa50. They further crossed each other at more than one point, revealing trivial boundaries between viscous and elastic characteristics. Depending on the monomer percentage, increasing the BisGMA ratio in such composite matrices could promote elasticity, while EgGAA addition would develop a viscous property of the composites. The difference in the overall patterns of moduli, in which either BisGMA or EgGAA dominated one another and changed from U- to S-like shapes, respectively, were seen. The moduli decreased with frequency increases from 0.1 to 0.4 rad/s in F-TBEa0 and F-TBEa25, followed by a frequency-independent stage up to 6.4 rad/s. Finally, with increasing shear frequency for all composites, they gradually increased.

### 3.5. Degree of Conversion Analysis

The degree of conversion (DC) of F-TBEa composites was determined via the ATR-FTIR technique [17]. Table 2 shows a decrease in DC with increasing EgGAA content, with no significant difference up to F-TBEa50; thus, the third hypothesis is partially supported. However, as EgGAA totally replaced BisGMA, the difference in DC became substantial, and the third hypothesis was then rejected. The DC of the control (F-TBEa0, EgGAA-free composite) was the highest (61.22%) compared to EgGAA-containing composites, i.e., F-TBEa25 (59.85%), F-TBEa50 (59.50%), and F-TBEa100 (52.54%). Hence, the DC of F-TBEa0–F-TBEa50 composites were above the clinically recommended value of >55%, whereas the value of F-TBEa100 in which the EgGAA totally replaced BisGMA, was below the minimum acceptable value. Thus, the possible incorporation of EgGAA up to 25 wt% of the organic matrix (F-TBEa50) is suggested. The reduced DC could be attributed to the lower viscosity of EgGAA compared to BisGMA, as seen in Table 2. As a consequence, the viscoelasticity of the composite, expressed in terms of complex viscosity, was reduced accordingly with EgGAA content increase. It is worth mentioning that both the low viscosity of EgGAA and the free radical inhibitory effect of Eg-moiety may play the major role on DC of EgGAA-containing composites. Hence, a competition between the two influencers is expected. This may be the case of balancing DC of F-TBEa25 and F-TBEa50 close to the control, despite viscosity differences. The development of the composite viscous portion due to the addition of EgGAA could enhance the mobility of radicals, resulting in DC increase; in contrast, the EgGAA raises the Eg-moiety in the matrix, possibly retaining some radical scavenging properties of Eg and resulting in an elevated inhibitory effect. Hence, the significant drop in the DC of F-TBEa100 could be attributed to the Eg-based free radical inhibition; this may dominate the advantage of viscosity reduction of the composite.

## 4. Conclusions

Dimethacrylated eugenol derivative (EgGAA) was successfully synthesized by following two sequenced reactions from eugenol, glycidyl methacrylate, and acryloyl chloride precursors in the presence of organic base catalysts, under an inert environment. Spectra analysis affirmed, through various techniques, its structural integrity as a difunctional monomer. It was found to be less thermally stable due to losing one methacrylate functionality below 300 °C. It has a glass transition temperature of −46.68 °C (DSC) and a viscosity of 0.379 Pa·s (rheometry). Its application in place of BisGMA in a resin matrix containing TEGDMA diluent can significantly reduce the viscosity of the resin, e.g., a reduction of up to 83 times after total replacement of BisGMA with EgGAA (TBEa0 = 0.164; TBEa100 = 0.010 Pa·s). Composites containing 66 wt% nanosilica revealed non-Newtonian, shear thinning behavior and a higher viscous portion when EgGAA replaced BisGMA in the matrix. The DC was not affected by EgGAA replacement of BisGMA up to F-TBEa50, beyond which, the replacement may become unfavored. Therefore, with its promising structural properties, this new difunctional monomer (EgGAA) could be considered for the subsequent development of resin-based composites.

## Figures and Tables

**Table 1:** Composition of matrices (TBEg (0–100)) and composites (F-TBEg (0–100)). Composites (F-TBEa0–F-TBEa100) consist of matrix and filler, matrices (TBEa0–TBEa100) combine base and diluent monomers, and the base monomers are either BisGMA or EgGAA or their mix.

Composite Code	Matrix, 34 wt%							Filler, 66 wt%
	Resin Code	Diluent		Base				
		TEGDMA		BisGMA		EgGAA		
		wt%	mol%	wt%	mol%	wt%	mol%	
F-TBEa0	TBEa0	50.00	64.16	50.00	35.84	0.00	0.00	Synthesized nanosilica (S-SiO_2_)
F-TBEa25	TBEa25	50.00	62.11	37.50	26.02	12.50	11.87	
F-TBEa50	TBEa50	50.00	60.18	25.00	16.81	25.00	23.01	
F-TBEa100	TBEa100	50.00	41.84	0.00	0.00	50.00	58.16	

**Table 2:** Rheological results for monomers (BisGMA, UDMA, EgGAA, and TEGDMA), unfilled resins (TBEa0–TBEa100), and silica filled resin-based composites (F-TBEa0–F-TBEa100), and degree of conversion (DC) of filled composites.

Rheological Results									Degree of Conversion (DC; %), *n* = 5	
Viscosity (Pa·s), *n* = 3						Complex Viscosity (Pa·s), *n* = 3				
Monomer			Resin			Composite				
Monomer Code	Av.	SD	Resin Code	Av.	SD	Composite Code	At 1.0 (rad/s); Av.	At 1.0 (rad/s); SD	Av.	SD
BisGMA	580.977 ^A^	12.648	TBEa0	0.164 ^A^	0.034	F–TBEa0	174.553 ^A^	20.917	61.22 ^A,C^	3.05
UDMA	9.106 ^B^	0.530	TBEa25	0.074 ^B^	0.002	F–TBEa25	102.726 ^B^	6.715	59.85 ^A^	2.44
EgGAA	0.379 ^B^	0.014	TBEa50	0.042 ^C^	0.002	F–TBEa50	92.543 ^B^	23.248	59.50 ^A^	3.07
TEGDMA	0.003 ^B^	0.000	TBEa100	0.010 ^D^	0.002	F–TBEa100	66.269 ^B^	5.990	52.54 ^B^	5.95

According to one-way ANOVA and Tukey post-hoc. Within the column: different uppercase letters denote statistical differences.

## Data Availability

The data used to support the findings of this study are included within the article.

## Supplementary Materials

The following supporting information can be downloaded at: https://www.mdpi.com/article/10.3390/polym15061481/s1, Section S1: Reaction Mechanism; Figure S1: Proposed reaction mechanisms for synthesizing EgGMA (reaction 1) and EgGAA (reaction 2); Figure S2: Proposed mass fragmentation route of EgGAA monomer; Figure S3: Chemical structure of BisGMA, UDMA, TEGDMA and EgGAA monomers. Table S1: FTIR bands of Eg, GMA, EgGMA, and EgGAA monomers; Table S2: ^1^H- and ^13^C NMR peaks assignments of Eg, GMA, EgGMA, and EgGAA; Table S3: Experimental and calculated mass the fragments of molecular ion; Table S4: Thermal decomposition of EgGAA monomer; Table S5: DSC data of BisGMA, TEGDMA and EgGAA monomers.
