# Supplementary material for: Novel 1,2-Bismethacrylate-3-Eugenyl Propane for Resin Composites: Synthesis, Characterization, Rheological, and Degree of Conversion
Source: Polymers (Basel). 2023 Mar 16;15(6):1481. doi: 10.3390/polym15061481 (PMC10053438; doi:10.3390/polym15061481)
Supplement: Supplementary file 1 [file polymers-15-01481-s001.zip › polymers-2275083-supplementary.pdf]

Suuplementary

Novel 1,2-Bismethacrylate-3-Eugenyl Propane for Resin Composites: Synthesis, Characterization, Rheological, and Degree of Conversion

Haifa Masfeer Al-Kahtani [1], Abdel-Basit Al-Odayni [2,3,*], Waseem Sharaf Saeed [2], Ali Robaian [4], Abdullah Al-Kahtani [1], Taieb Aouak [1] and Ali Alrahlah [2,5,]

1 Chemistry Department, College of Science, King Saud University, P.O. Box 2455, Riyadh 11451, Saudi Arabia

2 Engineer Abdullah Bugshan Research Chair for Dental and Oral Rehabilitation, College of Dentistry, King Saud University, Riyadh 11545, Saudi Arabia;

3 Department of Chemistry, Faculty of Education, Thamar University, Dhamar 87246, Yemen

4 Conservative Dental Sciences Department, College of Dentistry, Prince Sattam Bin Abdulaziz University, Alkharj 11942, Saudi Arabia

5 Restorative Dental Sciences Department, College of Dentistry, King Saud University, Riyadh 11545, Saudi Arabia

* Correspondence: aalodayni@ksu.edu.sa

S1. Reaction Mechanism

The reaction was carried out in two steps. The first step is the synthesis of EgGMA and the second is the synthesis of EgGAA from EgGMA, as shown in Figure S1.

S1.1. Synthesis of EgGMA

The synthesis of EgGMA could proceed via epoxy–ring opening followed by condensation reaction mechanism. First, Eg (1) molecule can be deprotonated by Ph_3P (2) organic catalyst to eugenol phenoxide intermediate (3), a step that facilitated by the nucleophilic properties of Ph_3P and stabilized by phosphonium ion Ph_3PH^+ (4). The nucleophilic properties of Eg-phenoxide (3) promotes epoxy ring opening through attacking either one of the two epoxy carbons in GMA (5), resulting in EgGMA alkoxide (6). Depending on which epoxy carbon was attacked by (3), the (6) anion can be either primary or secondary, however, secondary alkoxide is eventually favored due to a steric hindrance of the intermediate complex. Subsequently, this alkoxide (6) can exchange proton with phosphonium ion (4) into EgGMA (7) and regenerated catalyst. In this regard, catalyst could recycle and may function again on new Eg molecule; however, catalyst turnover was not examined. It is important to note that alternative mechanism steps may occur. For instance, phosphene (2) may attack GMA epoxy carbons (5) first, then an intermediate complex of GMA-phosphonium ion is formed. Eg-phenoxide ion of Eg is one expected structure that known to be stabilized by resonance, thus the Eg deprotonation may be easily achieved by GMA-phosphonium complex, followed by substitution of phosphonium by Eg-phenoxide ion to end up with EgGMA (7).

S1.2. Synthesis of EgGAA

The dimethacrylated Eg-derivative was synthesized through an addition-elimination reaction, a nucleophilic substitution mechanism, between MAC and EgGMA to produce ester. First, secondary alcohol molecule (7) acts as a nucleophile (or electron pair donor) and attack the partially positive carbon of carbonyl in MAC (8), while, simultaneously, the pi–pair of carbonyl moves onto the oxygen atom to give complex (9). Next, complex (9) undergoes deprotonation, facilitated by Et_3N (10) catalyst and resulting in a highly unstable ionic intermediate (11) in which the chlorin atom pulls bond electrons and leaves as chloride ion. The expelled Cl^- could react with ammonium ion (12) into (13) and, simultaneously, the C=O bond reformed by shifting of electrons from negative oxygen to give the final ester EgGAA (14).

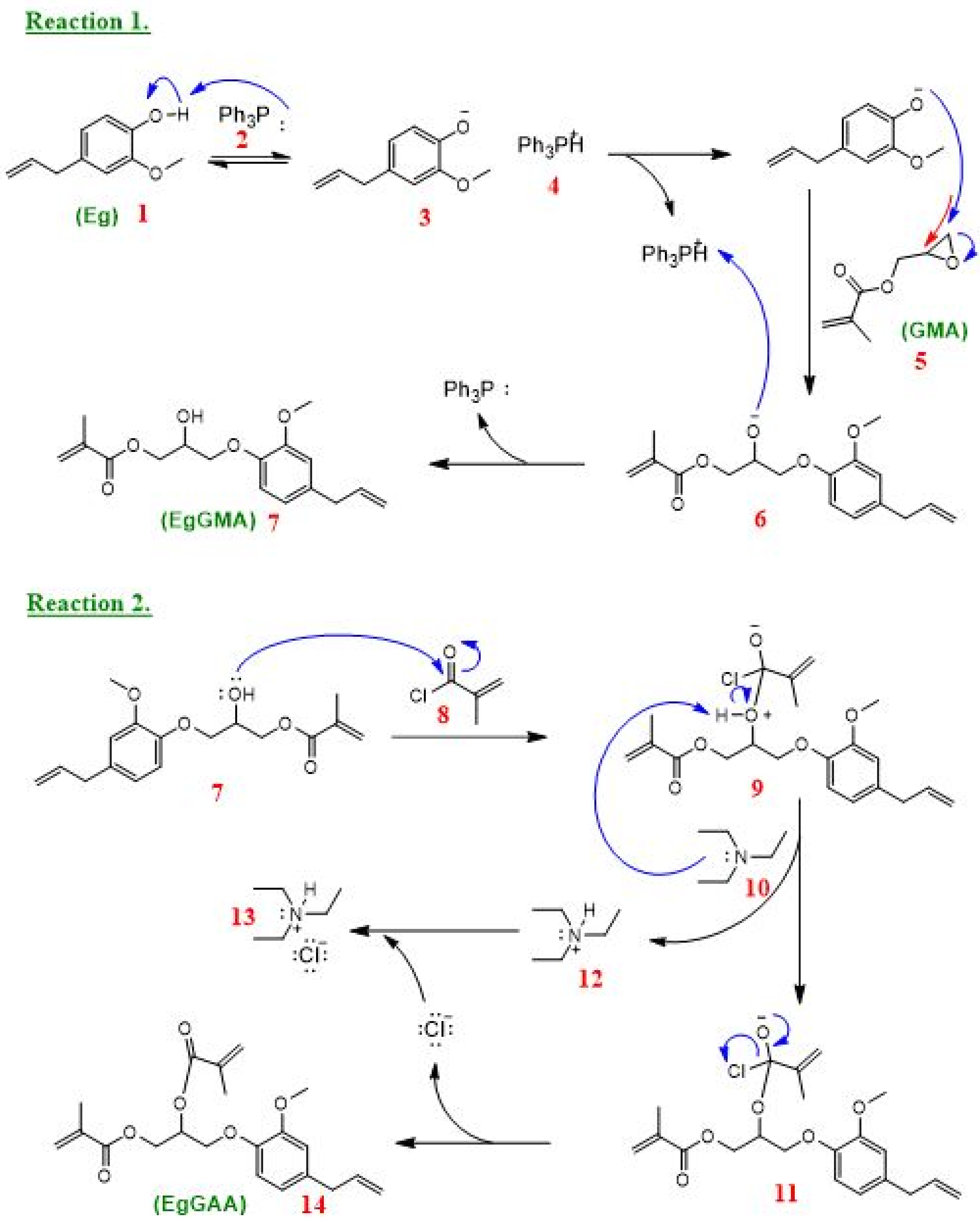

Figure S1. Proposed reaction mechanisms for synthesizing EgGMA (reaction 1) and EgGAA (reaction 2).

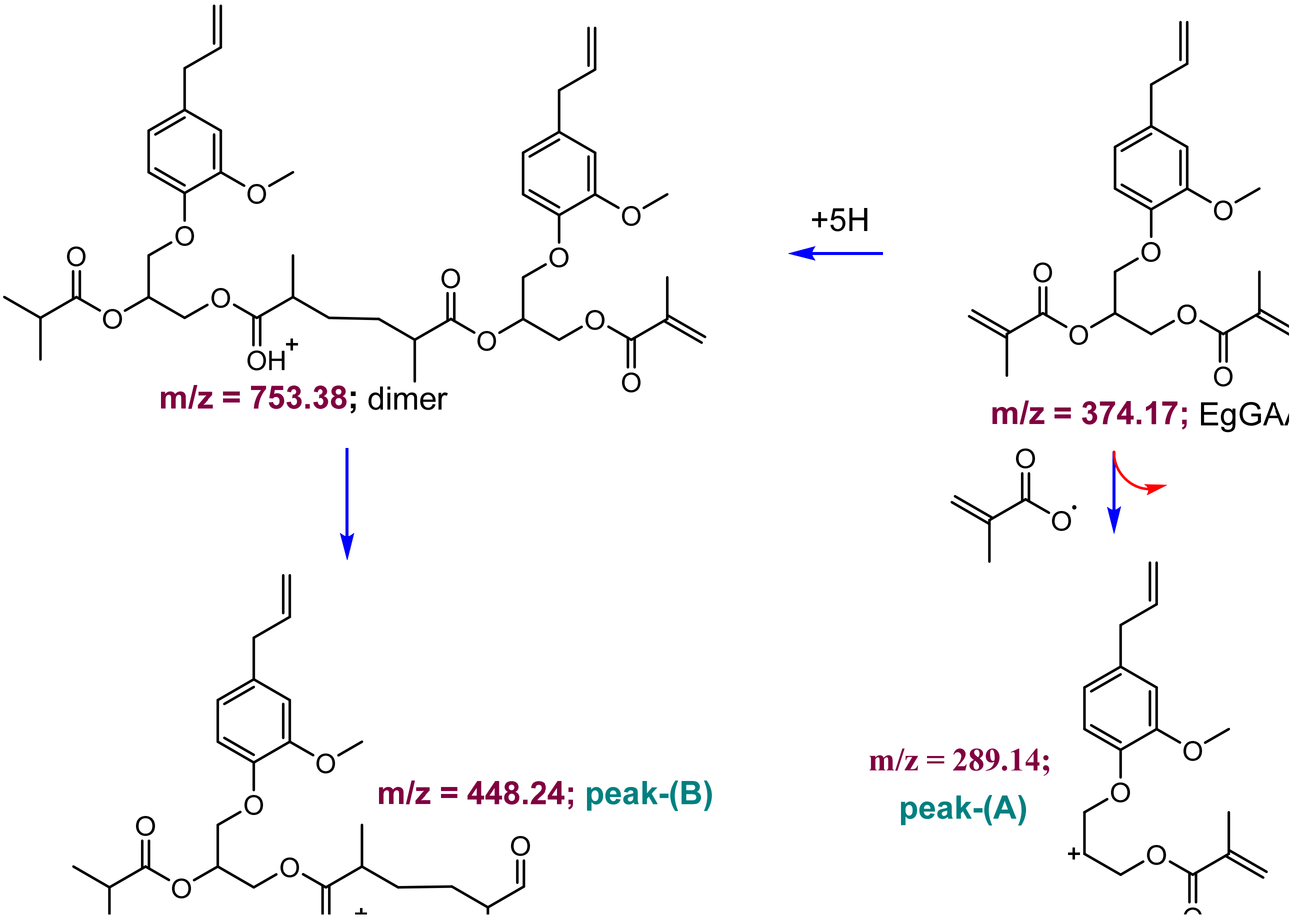

Figure S2. Proposed mass fragmentation route of EgGAA monomer.

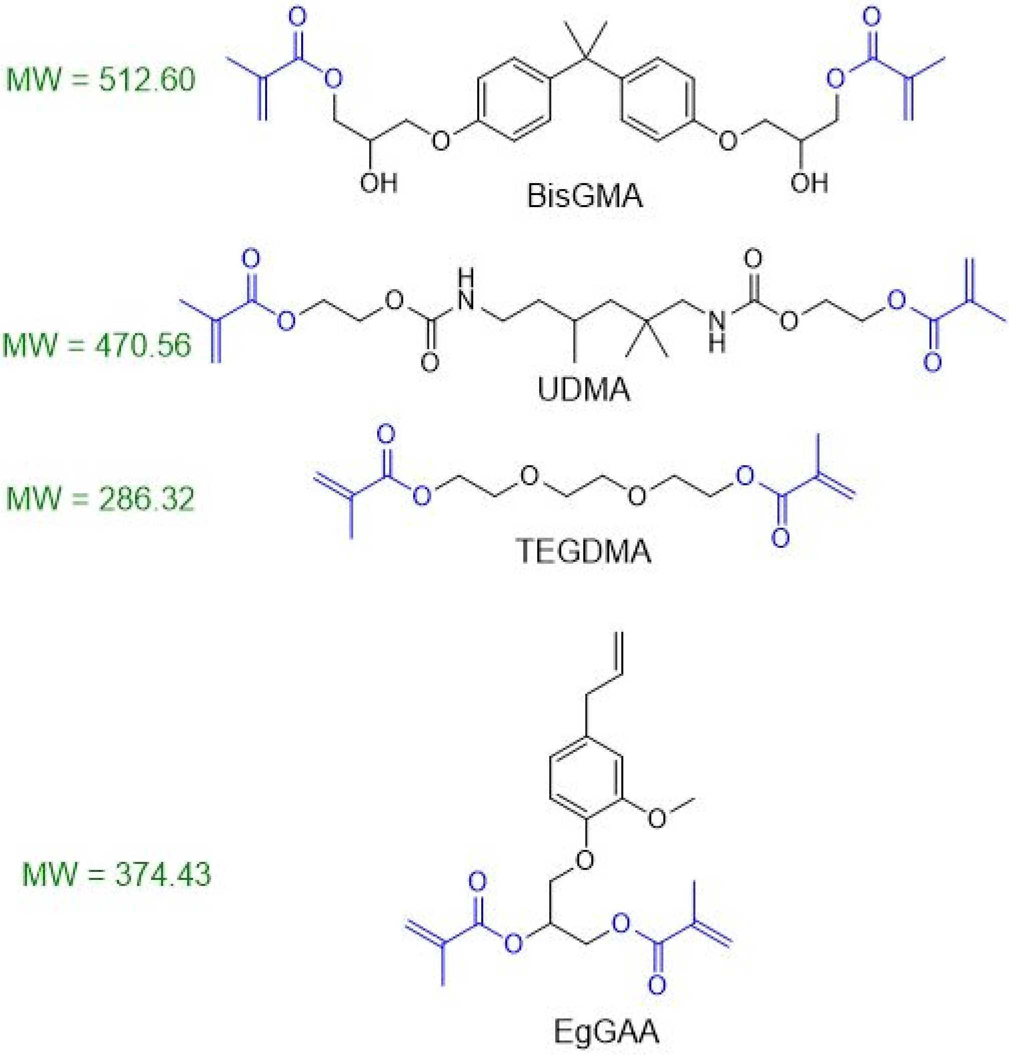

Figure S3. Chemical structure of BisGMA, UDMA, TEGDMA and EgGAA monomers.

Table S1. FTIR bands of Eg, GMA, EgGMA, and EgGAA monomers.

Peak assignment	Eg (cm^{-1})	GMA (cm^{-1})	EgGMA (cm^{-1})	EgGAA (cm^{-1})
ν(O-H)	3515, b; 3451, b	-	3436, b	-
ν(=C-H)	3076, w; 3003, w	3060, w; 3001, w	3077, w;	3082
ν(-C-H)	2975, 2938, 2905, 2842, w	2958, 2931, w	2955, 2932, 2836, w	2985, 2955, 2931, 2846, w
ν(C=O)	-	1716, vs	1716, vs	1759, sh; 1721, vs; 1677, s (may be for H-bonding??)
ν(C=C) aliphatic	1638, s	1638, s	1637, s	1638, m
ν(C=C), aromatic	1612, 1606, s	-	1606, w; 1591, m	1606, w; 1592, m
$\delta_{as}(CH_3)$	1511, vs	1515, w	1511, vs	1510, s
$\delta(CH_2)$	1463, m	1482, w	1466, w	1463, sh
$\delta_{as}(CH_3)$	1451, m	1453, m	1452, m	1451, s
RB, $\delta_s(CH_3)$	1431, m	1436, 1403	1434, vw, 1419, w	1419, w;

RB, δ_{ip}(COH)	1366, m	1379, 1349, m	1378, m	1377, m; 1320, m
RB, ν(C-O)	1265, s; 1231, s	1294, 1255, m	1295, 1230, m	1286, 1263, 1230, m
τ(CH_2); ϱ_{ip}(CH_3); ϱ_{op}(CH_3); δ_{ip}(CH)	1204, s; 1181, s; 1148, s	1154, vs	1156, 1140, s	1151, s;
RB,	1121	-	Overlapped	1123, m
ν(C-O)	1033	1078, w	1032	1109, m
τ(C=C)	994,	1016, m	994, w	994, w
δ_{op}(CH)	947,	942, m	944, m	945, s
ν(C-O)	912, s	907, Epoxy ring	914, m	912, m
δ_{op}(CH)	850, m; 816, m	880–650, m	850, m; 799, m	851, m; 812, s;
RB	793, m; 745, m; 720, m	-	747, 651, 618, m	748–613, m

Abbreviation: Vibration type: δ, symmetric bending vibration; ω, wagging; ϱ, rocking vibration; ν = symmetric stretching; τ, twisting. Peak strength: b, broad; m, medium; s, strong; sh, shoulder; vs, very strong; vw, very weak; w, weak.

Table S2. ^{1}H- and ^{13}C NMR peaks assignments of Eg, GMA, EgGMA, and EgGAA.

**^{1}H**	^{1}H NMR				^{13}C	^{13}C NMR			
	Eg	GMA	EgGMA	EgGAA		Eg	GMA	EgGMA	EgGAA
**a**	-	6.07 (s, 1H	6.11 (s, 1H)	6.09 (d, J = 13.2 Hz, 2H)	1	-	125.96	126.05	127.73
a'	-	5.53 (d, J = 1.8 Hz, 1H),	5.56 (s, 1H)	5.63-5.50 (m, 2H),	2	-	135.89-135.34	136.18-135.52	136.54
b	-	1.87 (s, 3H)	1.92 (s, 3H)	1.92 (s, H)	3	-	18.03	18.92	17.48
c	-	2.83-2.70 (m, 1H),	4.12-3.91 (m, 2H)	4.31-4.20 (m, 1H),	4	-	166.77	167.37	167.32
c'	-	2.65-2.52 (m, 1H),		4.10-3.93 (m, 1H),	5	-	64.96	67.68	68.96
d	-	3.17 (q, J = 2.9 Hz, 1H),	5.21 (d, J = 5.1 Hz, 0H),	5.71 (m, 1H)	6	-	49.15	65.32	65.21
e	-	4.39 (dt, J = 12.2, 2.7 Hz, 1H),	4.37-4.15 (m, 3H)	4.31-4.20 (m, 2H),	7	143.80	-	146.25	146.04
e'	-	3.91 (qd, J = 6.1, 2.6 Hz, 1H)			8	146.37	-	149.86	149.61
f	6.88 (d, J = 8.8 Hz, 1H)	-	6.84 (q, J = 3.9 Hz, 1H)	6.83 (d, J = 8.8 Hz, 1H),	9	111.04	-	116.87-115.47	116.29-115.41
g	6.71 (t, J = 2.2 Hz, 2H)	-	6.69 (t, J = 3.3 Hz, 2H)	6.69 (d, J = 5.9 Hz, 2H)	10	131.82	-	134.48	134.18
h		-			11	121.07	-	121.84-120.95	121.43
i	3.34 (d, J = 6.6 Hz, 2H)	-	3.31 (d, J = 6.6 Hz, 2H),	3.30 (d, J = 6.6 Hz, 2H),	12	114.22	-	113.12	111.55
i'		-			13	39.83	-	38.54	39.45
j	6.04-5.90 (m, 1H)	-	6.01-5.84 (1H)	5.97-5.85 (m, 1H)	14	137.76	-	136.64	138.08

k	5.17-5.02	-	5.13-4.97	5.12-4.97	15	115.35	-	117.28	117.19
k'	(m, 2H)	-	(m, 2H)	(m, 2H)	16	55.73	-	56.47	56.40
l	3.88 (s, 3H)		3.88-3.72 (m, 3H)	3.86-3.75 (m, 3H),	17	-	44.40	63.87	63.73

Table S3. Experimental and calculated mass the fragments of molecular ion.

**No.**	**Experimental mass**	**Calculated mass**	**Mass difference (mmu)**	**Formula**	**Unsaturation degree**
Calibration PEG: 1.21-1.36 min. 320-500 Da – 2.447.E-13					
1	374.17110	374.17294	-1.84	$C_{21}H_{26}O_6$	9.0
2	375.18129	375.18076	0.53	$C_{21}H_{27}O_6$	8.5
3	380.21853	380.21989	-1.36	$C_{21}H_{32}O_6$	6.0
4	381.22546	381.22771	-2.25	$C_{21}H_{33}O_6$	5.5
5	382.23373	382.23107	2.67	$C_{20}{}^{13}CH_{33}O_6$	5.5

Table S4. Thermal decomposition of EgGAA monomer.

**TGA**	**Temperature range (°C)**	**DTG_{max} (°C)**	Weight loss (%)	Assignment	
				Formula	Mass (%)
**Step 1**	**134–221**	**190**	18	C_4H_5O	18.7
Step 2	220-327	277	9	OCH_3	8.1
Step 3	327-517	427	60	backbone	-
Residue	800	-	11	3C	10

Table S5. DSC data of BisGMA, TEGDMA and EgGAA monomers.

**Monomer**	**Onset (°C)**	**Endset (°C)**	**Mid-point (°C)**
BisGMA	−12.05	−7.38	-9.65
EgGAA	−50.06	−43.61	-46.68
TEGDMA	−88.57	−85.14	-86.85
